# Compound Retention in Care and All-Cause Mortality Among Persons Living With Human Immunodeficiency Virus

**DOI:** 10.1093/ofid/ofz120

**Published:** 2019-03-07

**Authors:** Emma Sophia Kay, D Scott Batey, Andrew O Westfall, Katerina Christopoulos, Stephen R Cole, Elvin H Geng, W Christopher Mathews, Richard D Moore, Michael J Mugavero

**Affiliations:** 1 University of Michigan, Ann Arbor; 2 University of Alabama at Birmingham; 3 University of California, San Francisco; 6 University of California, San Diego; 4 University of North Carolina at Chapel Hill; 5 Johns Hopkins University, Baltimore, Maryland

**Keywords:** hazard ratio, HIV/AIDS, mortality hazards, retention in care

## Abstract

**Background:**

To obtain optimal health outcomes, persons living with human immunodeficiency virus (HIV) must be retained in clinical care. We examined the relationships between 4 possible combinations of 2 separate retention measures (missed visits and the Institute of Medicine [IOM] indicator) and all-cause mortality.

**Methods:**

The sample included 4162 antiretroviral therapy (ART)–naive patients who started ART between January 2000 and July 2010 at any of 5 US sites of the Center for AIDS Research Network of Integrated Clinical Systems. The independent variable of interest was retention, captured over the 12-month period after the initiation of ART. The study outcome, all-cause mortality 1 year after ART initiation, was determined by querying the Social Security Death Index or the National Death Index. We evaluated the associations of the 4 categories of retention with all-cause mortality, using the Cox proportional hazards models.

**Results:**

Ten percent of patients did not meet retention standards for either measure (hazard ratio [HR], 2.26; 95% confidence interval [CI], 1.59–3.21). Patients retained by the IOM but not the missed-visits measure (42%) had a higher HR for mortality (1.72; 95% CI, 1.33–2.21) than patients retained by both measures (41%). Patients retained by the missed-visits but not the IOM measure (6%) had the same mortality hazards as patients retained by both measures (HR, 1.01; 95% CI, .54–1.87).

**Conclusions:**

Missed visits within the first 12 months of ART initiation are a major risk factor for subsequent death. Incorporating missed visits in clinical and public health retention and viral suppression programming is advised.

## Teaser

Missed visits within the first 12 months after antiretroviral therapy initiation are a major risk factor for subsequent death in persons living with human immunodeficiency virus. Incorporating missed visits in clinical and public health retention and viral suppression programming is advised.

It is established that, to attain and maintain optimal health outcomes, persons living with human immunodeficiency virus (HIV) (PLWH) must be retained in clinical care [1–3]. In fact, retention in care (henceforth, “retention”) is 1 of 5 key steps in the HIV care continuum. This is a framework used to represent HIV care as a progression from testing to linkage and retention in care, prescription of antiretroviral therapy (ART), and, as the ultimate goal of ART and the preeminent biomarker of treatment success, viral suppression [4–6]. According to estimates from the Centers for Disease Control and Prevention (CDC), less than half (48%) of the 1.1 million PLWH in the United States were retained in care in 2014 [7]. This poses a health concern for PLWH as well as for population-level health, because PLWH who do not regularly attend their primary healthcare appointments are less likely to adhere to ART and, subsequently, are at increased risk of detectable virema with associated adverse health outcomes and increased sexual transmission risk [8]. Poor retention also has negative implications for clinics because the time slots and resources reserved for patients who did not attend their scheduled visits go unused [9].

Given that retention is crucial for personal and population health, it is paramount that this step in the HIV care continuum receives continued attention. The National HIV/AIDS Strategy (NHAS) seeks to increase the proportion of PLWH who are retained in care to 90% by 2020 [10]. To reach and maintain this ambitious national target, however, it is important that the retention health quality indicator be systematically captured and recorded as an ongoing prognostic measure of health. To this end, the retention indicator adopted by the Institute of Medicine (IOM) is endorsed by the NHAS as the metric to monitor retention [11, 12]. Notably, previous research has demonstrated that there is no gold standard to measure retention and that being retained in care, according to various commonly used retention measures, is associated with viral suppression [13, 14].

We previously evaluated the relationship between missed (“no-show”) primary care visits and increased mortality risk and found that, even when patients were classified as retained in care by the IOM indicator, those who missed ≥3 scheduled visits had a significantly higher risk of death than patients who did not miss any scheduled visits [14]. Yet, few studies have compared HIV retention indicators to each other, whether in isolation, used in combination, or sequentially. For instance, an analysis of 2811 PLWH in the Kaiser Permanente Northern California healthcare system found that patients with ≥1 missed visit in their first year after diagnosis had a 71% increased mortality risk compared with patients who did not miss any visits in that first year [15]. 

A Brazilian-based study found that patients who were new to HIV care and who missed more than a single 6-month visit during their first 2 years of care had an increased risk of death compared with patients who attended ≥3 of these 6-month visits [16]. Finally, research conducted in Kenya found that patients who missed a scheduled visit during a 12-month period had >6 times the risk of death of patients who did not miss any visits, and that mortality risk increased with the number of missed visits [17]. Although these studies help illustrate the link between missed visits and mortality risk, less is known about the clinical significance of retention on mortality when multiple retention measures are combined, which we call *compound retention*.

In the current study, we examined the relationship between the 4 possible combinations of all-cause mortality with 2 separate retention measures, missed visits and the IOM indicator. We hypothesized that each of the 4 possible combined retention categories (Table 1) would, when contrasted, be differentially associated with all-cause mortality. Our study aim was to examine the relationship between these retention measures captured over the 12-month period after ART initiation and all-cause mortality at the end of this period.

**Table 1.** All-Cause Mortality by Cross-Tabulation of Retention Measures

Retention per IOM Measure^a^	Retained per Missed-Visits Measure (0 No-Shows)	Not Retained per Missed-Visits Measure (≥1 No-Show)
Retained	All-cause mortality for patients retained by both measures	All-cause mortality for patients retained by IOM measure only
Not retained	All-cause mortality for patients retained by missed-visits measure only	All-cause mortality for patients not retained by either measure

Abbreviation: IOM, Institute of Medicine.

^a^The IOM measure defines retention as having ≥2 kept (attended) primary care visits separated by ≥90 days during a 12-month period.

## METHODS

### Sample

The sample consisted of 4162 ART-naive adult patients (≥18 years old) who initiated therapy between January 2000 and July 2010 at any of 5 sites associated with the Center for AIDS Research Network of Integrated Clinical Systems (CNICS), including the University of Alabama at Birmingham, the University of Washington, the University of California at San Diego, the University of North Carolina at Chapel Hill, and Case Western Reserve University. CNICS is a multisite clinical cohort that includes 8 geographically diverse HIV clinical sites across the United States [18]. All CNICS sites maintain electronic health record systems, thereby creating a centralized high-quality clinical database that captures clinical data from over 32 000 PLWH across sites [19, 20]. Although these data were collected at a time when ART regimens were comparatively less advanced, they offer an instructive glimpse into HIV care attendance patterns and associated mortality hazards.

Because we examined all-cause mortality for patients who had been taking ART for 1 year, patients who died during the 12 months after ART initiation (n = 224) were excluded because the observation window was incomplete. The study was approved by institutional review boards at all 5 participating sites.

### Variables of Interest

The primary independent variable was retention. We used 2 separate measures for retention captured over the 12-month period after patients’ initiation of ART. We used the IOM indicator endorsed by the NHAS for our *kept visits* measure, which defines retention as having ≥2 kept (or attended) primary care visits separated by ≥90 days during a 12-month period [11, 21]. We use the terms *kept visit* and *missed visit* to indicate the that the former measure looks at *attended* scheduled HIV primary care visits only, and the latter is a function of *missed* scheduled HIV primary care visits only.

A primary care visit was defined as a visit that addresses routine medical care and is scheduled in advance; this does not include emergency or walk-in visits or specialty care appointments. In keeping with prior research [13, 14], for the missed-visits measure, patients who missed no scheduled primary care visits during the 12-month observation window were considered retained, and patients who missed ≥1 visit during that period were not retained (visits that were cancelled by the patient or provider were omitted from this calculation). Patients who did not attend any scheduled visits during the study period were considered not retained by both measures. Sensitivity analyses were also conducted in which more liberal cutoffs of <2 and <3 missed (no-show) visits were used for dichotomization of retained according to the missed-visits retention measure (Table 1).

The outcome of interest, all-cause mortality, is recorded for all CNICS patients. CNICS sites regularly confirm and update patient all-cause mortality information by querying the Social Security Death Index and National Death Index twice a year. This way, CNICS can systematically capture and record reported deaths that were not already included in CNICS patient records. Even patients lost to follow-up are included in these queries and, if needed, their patient records are updated accordingly [22].

We also included the following clinical and sociodemographic measures as covariates based on characteristics associated with retention in the literature (eg, in [23]): CNICS site, age, race/ethnicity, sex, HIV transmission risk group (men who have sex with men, intravenous drug user, or heterosexual), and CD4^+^ cell counts and viral load values collected at the initial primary care visit, before ART initiation.

### Statistical Analysis

Descriptive statistics for independent and dependent variables including means, standard deviations (SDs), counts, and frequencies, were conducted. We used χ^2^ tests to assess the relationship between the 2 retention measures and to obtain frequencies and percentages for the number of patients who comprised each of the 4 possible categories generated by the cross-tabulation of the 2 retention measures. We then used Cox proportional hazards models to evaluate the association of the 4 categories of retention with all-cause mortality while controlling for study covariates, which were measured at ART initiation (to account for the fact that patients who are not retained during the 1-year period will probably not have values at the 1-year mark). The time origin for the survival analyses was 1 year after patients began ART, to accommodate comparison of retention measures. We right-censored patients administratively on 31 July 2012. Patients were observed for a mean (SD) of 4.8 (3.3) years, with a median of 4.3, a minimum of 0, and a maximum of 11.8 years from 1 year after ART start until death or administrative censor. The median of the upper quartile was 7.3 years, and the median of the lower quartile was 2.0 years. For inclusion in the sample, patients had to have ≥1 year of potential time receiving ART.

**Figure 1.**
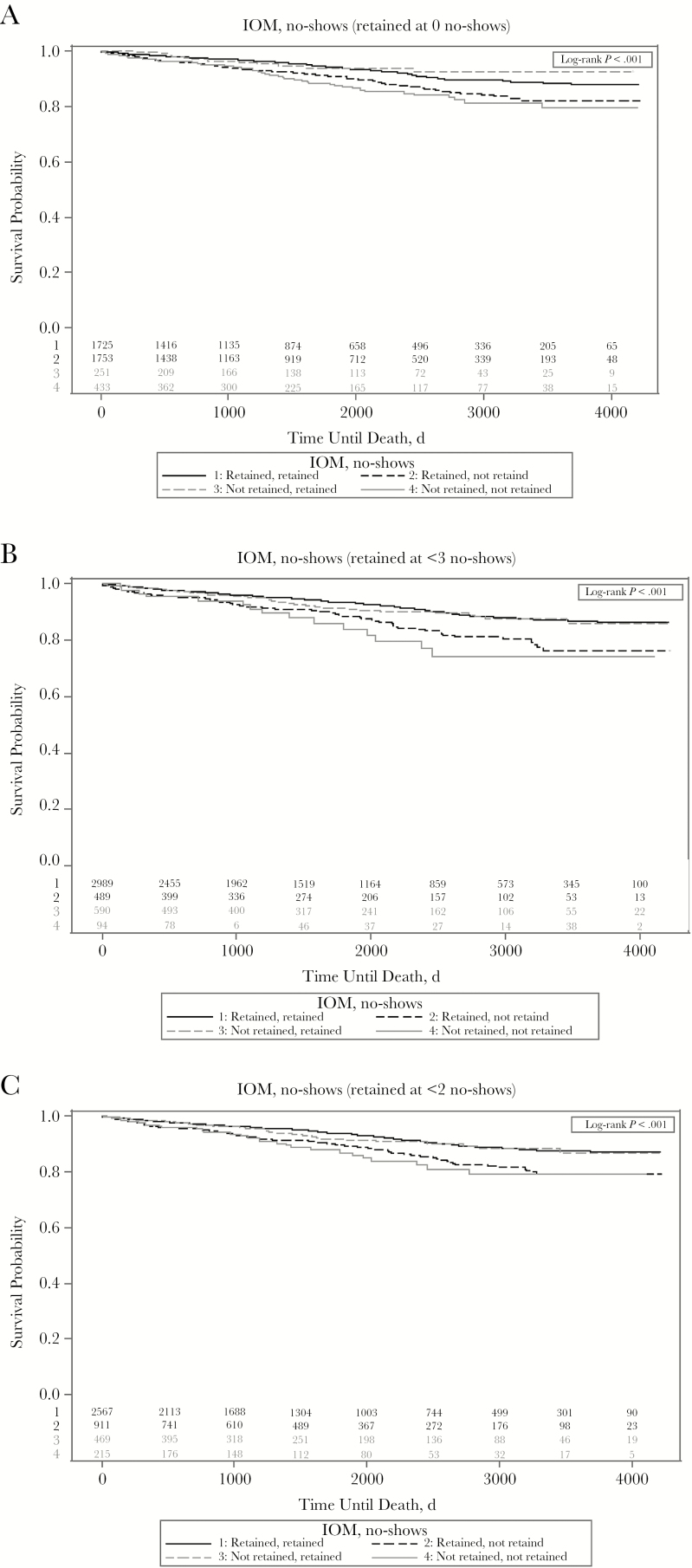
Kaplan–Meier survival curves for all-cause mortality among patients classified as retained or not retained 12 months after initiation of antiretroviral therapy according to Institute of Medicine (IOM) and missed-visits (no-show) indicators. (The IOM measure defines retention as having ≥2 kept [attended] primary care visits separated by ≥90 days during a 12-month period.) The number of patients at risk is shown above the x-axis. *A,* Retained defined as 0 no-shows. *B,* Retained defined as <3 no-shows. *C,* Retained defined as <2 no-shows.

We also examined all-cause mortality among patients classified as retained or not retained at 12 months after ART initiation for both retention measures, using Kaplan–Meier survival curves (Figure 1). Each patient’s first year of data was used to determine retention in care; beyond that, an individual patient’s data were not used (except for date of death). All analyses were conducted using SAS software, version 9.3 [24]. A significance level (α) of *P* < .05 was used to define statistical significance in all models.

## RESULTS

Most patients were male (80.7%), black (64.0%), and categorized in the “men who have sex with men” risk transmission group (55.1%), with a mean (SD) age of 38 years (10.2) years (Table 2). Most patients (83.2%) had viral loads >10 000 copies/mL and a CD4^+^ cell count <350/µL (74.5%) at baseline (at ART initiation).

**Table 2.** Selected Sample Characteristics

Characteristic	Patients, No. (%)^a^ (N = 4162)
Age, mean (SD), y	38.0 (10.2)
Sex	
Male	3360 (80.7)
Female	802 (19.2)
Race	
Black	1533 (64.0)
White	2227 (29.9)
Other/unknown	402 (6.1)
Risk transmission group^b^	
MSM	2241 (55.1)
Intravenous drug user	502 (12.4)
Heterosexual	1323 (32.5)
Baseline plasma HIV RNA^c^	
1000–10 000 copies/mL	559 (13.5)
10 001–100 000 copies/mL	1725 (41.4)
>100 000 copies/mL	1739 (41.8)
Baseline CD4^+^ T-lymphocyte count^d^	
<50/µL	869 (20.9)
50–199/µL	1027 (24.7)
200–349/µL	1201 (28.9)
350–500/µL	619 (14.9)
>500/µL	342 (8.2)

Abbreviations: HIV, human immunodeficiency virus; MSM, men who have sex with men; SD, standard deviation.

^a^Data represent no. (%) of patients unless otherwise specified. Percentages based on non-missing data only.

^b^Data missing in 96 patients (2.3%).

^c^Data missing in 139 patients (3.3%)

^d^Data missing in 104 patients (2.5%).

In the first year of taking ART, approximately half of patients were categorized as either retained by both measures or not retained by both measures, as shown in the diagonal cross-tabulations in Table 3. Nearly half (41%) of patients were retained by both measures, yet the same proportion (42%) were also retained by the IOM but not the missed-visits measure. In contrast, just 6% of patients were retained by the missed-visits but not the IOM measure.

**Table 3.** Cross-Tabulation of Institute of Medicine and Missed-Visits Retention Measures^a^

Retention per IOM Measure^b^	Patients, No. (%)	
	Retained per Missed-Visits Measure (0 No-Shows)	Not Retained per Missed-Visits Measure (≥1 No-Show)
Retained	1725 (41)	1753 (42)
Not retained	251 (6)	433 (10)

Abbreviations: IOM, Institute of Medicine;

^a^Covariates include site, age, race/ethnicity, sex, human immunodeficiency virus transmission risk group, and pre–antiretroviral therapy CD4^+^ cell count and viral load.

^b^The IOM measure defines retention as having ≥2 kept (attended) primary care visits separated by ≥90 days during a 12-month period.

When the hazard ratios (HRs) were generated by Cox proportional hazards modeling (Table 4), a pattern of mortality hazards emerged. Specifically, patients who were retained by the IOM but not the missed-visits measure had the highest HR for mortality (1.72; 95% confidence interval [CI], 1.33–2.21) when compared with patients retained by both measures. Patients who were considered not retained by either measure similarly had elevated mortality hazards (HR, 2.26; 95% CI, 1.59–3.21). On the other hand, patients retained by the missed-visits measure but not the IOM measure had the same HR as patients retained by both measures (1.01; 95% CI, .54–1.87). Results of the sensitivity analyses were complementary, with larger effect sizes for missed visits on mortality when more missed visits were needed to be considered not retained (Table 5).

**Table 4.** Hazard Ratios for All-Cause Mortality by Cross-Tabulation of Institute of Medicine and Missed-Visits Retention Measures^a^

Retention per IOM Measure^b^	HR for All-Cause Mortality (95% CI)	
	Retained per Missed-Visits Measure (0 No-Shows)	Not Retained per Missed-Visits Measure (≥1No-Show)
Retained	Referent	1.72^c^ (1.33–2.21)
Not retained	1.01 (.54–1.87)	2.26^c^ (1.59–3.21)

Abbreviations: CI, confidence interval; HR, hazard ratio; IOM, Institute of Medicine.

^a^Covariates include site, age, race/ethnicity, sex, human immunodeficiency virus transmission risk group, and pre–antiretroviral therapy CD4^+^ cell count and viral load.

^b^The IOM measure defines retention as having ≥2 kept (attended) primary care visits separated by ≥90 days during a 12-month period.

^c^ *P* < .001.

**Table 5.** Sensitivity Analysis for Hazard Ratios for All-Cause Mortality by Cross-Tabulation of Institute of Medicine and Missed-Visits Retention Measures^a^

Retention per IOM Measure^b^	HR for All-Cause Mortality (95% CI)			
	Retained per Missed-Visits Measure (<2 No-Shows)	Not Retained per Missed-Visits Measure (≥2 No-Shows)	Retained per Missed-Visits Measure (<3 No-Shows)	Not Retained per Missed-Visits Measure (≥3 No-Shows)
Retained	Referent	1.87^c^ (1.45–2.42)	Referent	2.12^c^ (1.58–2.84)
Not retained	1.32 (.90–1.93)	2.40^c^ (1.58–3.64)	1.35 (.97–1.87)	2.84^c^ (1.67–4.84)

Abbreviations: CI, confidence interval; HR, hazard ratio; IOM, Institute of Medicine.

^a^Covariates include site, age, race/ethnicity, sex, human immunodeficiency virus transmission risk group, and pre–antiretroviral therapy CD4^+^ cell count and viral load.

^b^The IOM measure defines retention as having ≥2 kept (attended) primary care visits separated by ≥90 days during a 12-month period.

^c^ *P* < .001.

## DISCUSSION

The cross-tabulation of 2 commonly used measures of retention among ART initiators provides an instructive glimpse into the relative mortality hazards experienced by PLWH who are classified into 1 of 4 retention categories in their first year of taking ART. Being classified as *not* retained by 1 of the 2 measures had a significant association with all-cause mortality, but only when that measure was based on missed visits. In fact, patients who were retained by the missed-visits measure, but not the IOM measure, had approximately the same mortality hazards as those who were retained by both measures.

These findings contribute to the literature that suggests that missed visits are associated with increased risk of death [14–17]. As a recent multisite analysis found that the strongest predictor of future missed visits is prior missed visits [25]; even a single missed visit may be indicative of poor HIV care attendance, and consequently poor HIV health, in the long term. Our findings also provide additional understanding about the relationship between multiple categories of retention, designated as *compound retention* in the current study, and all-cause mortality. Given that nearly half of the patients (42%) missed ≥1 visit during the 12 months after they started ART but were still classified as retained using the IOM kept visits measure, it is vital that clinicians make use of this missed-visits measure that is administratively and computationally straightforward. Indeed, if this analysis had measured retention using the IOM measure alone, the increased mortality hazards associated with missed visits would not have been apparent.

Using cross-tabulation, we found that combinations of retention measures are associated with differential mortality hazards for PLWH after their first year of taking ART. Clinicians can apply this tool to their practices, thereby identifying patients at risk who would benefit from targeted interventions aimed at increasing retention (to include both an increase in attended visits and a decrease in missed visits). There is evidence that even short, motivational interviewing-based retention strategies, such as the single-session, 60-minute intervention recently developed by Smith and colleagues [26], may be effective, even in fast-paced clinic settings [27].

Importantly, as has been observed elsewhere, there is no reference standard to measure retention [13, 14]. Although the IOM indicator did not independently predict mortality hazards among ART initiators in this clinical cohort study, this measure has clear value. Beyond being associated with viral suppression [28], the IOM indicator can be captured from public health surveillance data and used as a proxy for care visits, because viral load is routinely measured at HIV primary care visits. As such, it represents a benchmark and public health quality indicator, well served for application by the IOM and NHAS. Moreover, 2 visits over 12 months separated by ≥90 days, as captured by the IOM measure, may be considered a bare minimum of attended visits to constitute care retention. As increasing proportions of PLWH are being scheduled every 6 months, this measure still has clinical validity and utility. However, as suggested by our findings, other complementary retention measures, such as missed visits, have added prognostic value and, in certain settings, like HIV clinics, can be a valuable adjuvant to the IOM indicator. In particular, the missed-visits retention measure is instructive as an immediately actionable indicator of mortality hazards.

This research has several limitations. Foremost, the present report is an observational study and there may be unmeasured causes of death that differ between persons classified as retained and those classified as not retained. Such potential differential assessments of these causes may cause confounding. Although our sample represented 5 different geographically diverse sites, the results may not be generalizable to all PLWH in the United States. Because the outcome of interest was all-cause mortality, we were unable to discern whether death was caused by AIDS-related causes. Similarly, our outcome of interest, all-cause mortality, was determined using the Social Security Death Index and National Death Index, which may not account for all deaths. We did not systematically capture whether patients transferred care during the 12 months after they began ART, although it is likely that the transfer rate was minimal during the short observation window. In addition, these results cannot be generalized to PLWH who receive ART outside an HIV clinic setting. Finally, this research did not capture trends in retention over time, because retention outcomes were assessed only for the first year after ART initiation. However, proximal retention in care—especially that based on missed visits—is a valuable indicator available to clinicians that may help prevent premature death.

In conclusion, these results indicate that patients starting ART who miss scheduled visits during the following 12-month period, even if they are considered retained by IOM and NHAS standards, have significantly higher risks of death than those who do not have any no-show visits. Although the missed-visits measure seems to carry more clinical value, we still recommend use of the IOM measure for 2 reasons. First, many clinics do not collect detailed data on missed visits and keep records only on attended visits. Second, the IOM measure is the only retention measure that, thus far, has received national endorsement (ie, National HIV/AIDS Strategy and IOM).

If we are to reach 90% retention among PLWH in the United States by 2020 [10], substantial strides must be made to keep patients retained in care. Increasing retention among PLWH has individual-level as well as public health implications, as recently seen in the “U = U” campaign, which highlights the fact that having a sustained undetectable viral load effectively prevents sexual transmission [29]. To this end, incorporating missed visits in clinical and public health retention and viral suppression programming is advised based on study findings.
